# SMART Brain: Study of Menopause and Resistance Training on Brain Health

**DOI:** 10.1093/geroni/igaf122.1802

**Published:** 2025-12-31

**Authors:** Cindy Barha

**Affiliations:** University of Calgary, Calgary, Alberta, Canada

## Abstract

Two-thirds of all persons with dementia are females and females have a 2-fold greater lifetime risk of developing dementia than males. Exercise is a promising lifestyle intervention for promotion of cognitive function. We have shown that this is particularly true for older, postmenopausal females compared with older males. Perimenopause – one of the most significant endocrine transitions in a female’s life – negatively impacts the female brain and is believed to be a key contributor to a female’s greater lifetime risk for dementia. Thus, perimenopause may be a critical window to implement exercise interventions for the promotion of female brain health. There is a dearth of randomized controlled trials that examine the impact of exercise of any type on brain health in midlife (i.e., 40 to 60 years of age). Resistance training, specifically, may provide multi-system benefits for females as they transition through menopause. In a small pilot study, 35 perimenopausal and early postmenopausal participants underwent a 2x/week, 9-month resistance training intervention. Cognition was measured with the NIH Toolbox, a valid neuropsychological test battery, and task-induced hemodynamic response in the brain was measured using functional near infrared spectroscopy at baseline and trial-completion. Compared with the waitlist control group, a significant improvement was seen in cognitive flexibility in the intervention group. Non-significant improvements were also seen in the intervention group in processing speed, inhibitory control and verbal episodic memory delayed recall. These findings indicate that resistance training is a promising intervention for promoting cognition in midlife.

